# Renal failure in critically ill patients, beware of applying (central venous) pressure on the kidney

**DOI:** 10.1186/s13613-018-0439-x

**Published:** 2018-09-20

**Authors:** Xiukai Chen, Xiaoting Wang, Patrick M. Honore, Herbert D. Spapen, Dawei Liu

**Affiliations:** 1 Pittsburgh Heart, Lung, Blood and Vascular Medicine Institute, University of Pittsburgh, 200 Lothrop Street, BST E1240, Pittsburgh, PA 15261 USA; 2 Department of Critical Care Medicine, Peking Union Medical College Hospital, Peking Union Medical College, Chinese Academy of Medical Sciences, 1 Shuaifuyuan, Dongcheng District, Beijing, 100073 China; 3 Department of Intensive Care, Centre Hospitalier Universitaire Brugmann, Brugmann University Hospital, 4 Place Van Gehuchtenplein, 1020 Brussels, Belgium; 4 Department of Intensive Care, University Hospital, Vrije Universiteit Brussel (VUB), 101, Laarbeeklaan, Jette 1090 Brussels, Belgium

**Keywords:** Acute kidney injury, Central venous pressure, Afterload

## Abstract

The central venous pressure (CVP) is traditionally used as a surrogate of intravascular volume. CVP measurements therefore are often applied at the bedside to guide fluid administration in postoperative and critically ill patients. Pursuing high CVP levels has recently been challenged. A high CVP might impede venous return to the heart and disturb microcirculatory blood flow which may cause tissue congestion and organ failure. By imposing an increased “afterload” on the kidney, an elevated CVP will particularly harm kidney hemodynamics and promote acute kidney injury (AKI) even in the absence of volume overload. Maintaining the lowest possible CVP should become routine to prevent and treat AKI, especially when associated with septic shock, cardiac surgery, mechanical ventilation, and intra-abdominal hypertension.

## Background

Acute kidney injury (AKI) is a common complication in critically ill patients with high attributable morbidity and mortality [1, 2]. Systemic and renal perfusion considerably determines the development and course of AKI. Yet, optimal hemodynamic targets to minimize the risk of AKI are not precisely defined [3, 4]. In critical care, hypotension and shock are the “rogue enemies.” Resuscitation primarily focuses on optimizing mean arterial pressure (MAP) to improve renal perfusion [5]. However, there is little evidence that MAP correctly reflects organ perfusion. Moreover, aggressive fluid loading may contribute to an increased central venous pressure (CVP). By accepting high CVP levels [6–10], clinicians neglect that volume treatment and AKI are closely intertwined.

CVP is traditionally used for assessing volume status and volume responsiveness at the bedside [11]. However, CVP measurements to direct volume management in critically ill patients have repeatedly been found unreliable [12]. Whether and how CVP monitoring should be adapted to a particular patient (e.g., postsurgical, cardiac, septic) population is topic of controversy and debate [13, 14]. Monitoring CVP also does not guarantee preservation of renal function. A recent study reported a higher incidence of AKI in patients undergoing CVP monitoring as compared with unmonitored subjects. A 1 cm H_2_O higher CVP was associated with a 1.02 (95% CI 1.00–1.03, *p* = 0.02) risk of AKI. No association was found between pulmonary edema and AKI [13]. Till recently, the innate pressure character of CVP and its pathophysiological impact have been largely underestimated. What follows is a thorough discussion about the role of CVP, beyond its value as volume indicator, in various diseases.

## Main text

### CVP is a pressure used to estimate volume

The CVP is the pressure recorded from the superior vena cava or right atrium which, in the absence of tricuspid stenosis, equals right ventricular end-diastolic pressure. CVP is determined by the interaction between cardiac function and venous return which both depend on changes in total blood volume, vascular tone, cardiac output (CO), right ventricular compliance, intrathoracic and pericardial pressure [15]. CVP measurements are especially useful when followed over time and combined with a CO recording. A properly measured CVP can successfully guide right ventricular filling [16]. Within a certain range, CVP increases with expanding blood volume. However, excessive fluid administration may augment CVP and end-diastolic pressure without increasing end-diastolic or stroke volume. On the other hand, an increased CVP is often associated with decreased right ventricular compliance. Additionally, CVP is the downstream pressure for venous return and close to the minimum pressure in the global circulation [17].

### CVP and kidney “afterload”

CVP must be lower than renal venous pressure (RVP) in order to allow an adequate venous renal blood flow (RBF) to the heart. Accordingly, the presence of a high CVP requires a much higher RVP to ensure this flow. Renal perfusion pressure (RPP) approximates the difference between renal arterial pressure and RVP. As such, a higher RVP lowers RPP. In analogy with cardiac physiology, this forms the basis for the renal “afterload” concept [18]. Recent studies focusing on kidney “afterload” have revived interest in older studies which suggested that kidney dysfunction resulted from venous congestion transmitted to the renal venous compartment. Almost a century ago, it was indeed demonstrated that an hypervolemia-induced increase in RVP caused AKI independently of CO or RBF [19].

### Effect of CVP on pressure and flow in the kidney

Kidney perfusion is pressure and flow dependent. If intravascular volume augments without excessive CVP elevation, the unstressed volume (i.e., the fluid volume to fill the vascular bed to the point where it exerts force on the vessel walls) may incrementally follow a CO increase and RBF will rise. When CVP is already high, however, any additional volume load may increase CVP without a subsequent increase in CO and RBF. Right ventricular function then may deteriorate and evolve into acute cor pulmonale [17]. The difference between mean system filling pressure (MSFP) and CVP is the driving force behind venous return. Thus, with increasing CVP, a venous return will drop [20, 21]. With the heart functioning on the steep portion of the Starling curve, volume expansion will increase MSFP more than CVP. In contrast, changes in MSFP are approximately similar to CVP changes on the flat part of the Starling curve with no or minimal effects on CO [22, 23]. If fluid administration fails to obtain a higher MSFP, CVP must be kept low to enhance venous return, cardiac preload and CO. In isolated kidneys of healthy dogs, renal venous and tissue pressures were unaffected over a large range of increased venous pressures. However, RBF fell when RVP approached or exceeded renal venous and tissue pressure [24]. Critically ill patients even have a more narrow pressure autoregulation range [25]. In the cardiorenal syndrome, an elevated CVP causes lowering of RPP below the kidney autoregulation threshold, resulting in pressure-dependent renal perfusion [26]. The rise in CVP is transmitted to the renal veins, sustains the cardiorenal syndrome, and induces a detrimental feedback loop via the renin–angiotensin–aldosterone and neuroendocrine pathways that leads to refractory heart failure. Worsening congestion also enhances sodium retention which exacerbates heart failure.

### CVP can be more than a volume “indicator”

Many studies report a weak relationship between CVP and blood volume. CVP itself or changes in CVP evolution over time also failed to predict the hemodynamic response to a fluid challenge or to correctly estimate cardiac filling. As a result, it was suggested to abandon CVP to guide fluid resuscitation in critically ill patients [11, 27]. However, a more thorough understanding of various parameters and variables (i.e., preload, measurements in fluid-filled systems, impact of respiration, physiological determinants of CVP, and the point on the tracing that best estimates cardiac preload) may revalue CVP as a reproducible indicator of cardiac preload [16]. This is best illustrated by looking at the relationship between CVP and AKI in cardiac disease and sepsis.

#### Heart failure and cardiorenal syndrome

Since pressure/volume relationships are largely determined by heart compliance, a high CVP indicates volume overload, cardiac dysfunction, or both [28, 29]. Traditionally, AKI in congestive heart failure or cardiorenal syndrome is attributed to a reduction in CO and MAP which elicits a series of neurohumoral events resulting in increased renal vascular resistance and decreased renal function [30]. The degree of AKI is closely associated with congestive venous “backward failure.” In 2557 patients who underwent right heart catheterization, Damman et al. found that an increased CVP was not only associated with impaired renal function but also independently related to all-cause mortality [31]. A study in patients with advanced decompensated heart failure showed that those with worsening renal function had a higher CVP on admission and after intensive medical therapy [32]. Worsening renal function occurred less frequently in patients in whom CVP was kept below 8 mmHg. An apparent potential of CVP for AKI risk stratification was noted across the spectrum of systemic blood pressure, pulmonary capillary wedge pressure, cardiac index, and estimated glomerular filtration rate [32]. In adults with chronic heart disease after biventricular repair, Ohuchi et al. found that a high CVP predicted kidney enlargement and abnormal intrarenal flow dynamics that were closely associated with severity of heart failure and with cardiovascular events [33]. Right ventricular dysfunction and increased CVP are frequently observed in cardiac surgery patients and may lead to congestive renal dysfunction [34]. Studies in patients with acute right ventricular failure suggest that a high CVP is associated with a marked reduction in RBF by increasing renal backward pressure [35, 36]. A strong relationship was observed between CVP and RBF in both acute and chronic heart failure. Reducing CVP markedly improved renal function [35]. Cardiovascular surgery patients with progressive AKI had greater diastolic perfusion pressure deficits as compared to patients without AKI progression. Almost 25% of the diastolic perfusion pressure deficit was due to an increase in CVP [36]. This underscores the strong relationship between back (renal venous) pressure and CVP in the development of AKI.

Taken together, more attention must be paid to the pressure effect of CVP in heart failure/cardiorenal syndrome, regardless of whether fluid overload is present or not.

#### Sepsis and septic shock

Based on the landmark article of Rivers et al. which highlighted a striking mortality benefit of early goal-directed therapy (EGDT) in severe sepsis and septic shock [37], the Surviving Sepsis Campaign guidelines endorsed a CVP of 8–12 mmHg (12–15 mmHg in mechanically ventilated patients) as a key resuscitation target [38]. However, fluid load after 72 h in the Rivers study was equally high (approximately 13.5 L) in the EGDT and control arm. A major drawback of the study was the lack of data on occurrence and incidence of AKI. Recently, EGDT was assessed in the multicenter ProCESS [39, 40], ARISE [41], and ProMISE [42] trials which all used a CVP target ≥ 8 mmHg for guiding fluid resuscitation. The results of these trials, while reporting an all-time low sepsis mortality, question the need to use all elements of EGDT or the need for protocolized care in general. Limited data suggest that EGDT does not improve incidence of AKI and outcome of patients with AKI [43]. A CVP > 8 mmHg decreased microcirculatory and renal blood flow and increased AKI and mortality risk [44]. After adjustment for fluid balance and positive end-expiratory pressure ventilation, a lower diastolic arterial pressure and an elevated CVP were found to correlate with a high AKI incidence in septic patients [45–48].

Overzealous fluid treatment may result in interstitial edema which may worsen AKI or hamper renal recovery [49]. This underscores the potential role of venous congestion as one of the factors potentially implicated in the pathogenesis of septic AKI. CVP-directed fluid resuscitation in septic shock might harm the kidney if the target point is not correctly determined. Consequently, conservative fluid management [44] and permissive hypofiltration (“unburdening” the kidney by providing early renal replacement therapy, avoiding new injurious events such as fluid overload, and initiating therapies to improve survival and avoid ongoing loss of kidney function) [50, 51] are emerging treatment options in septic AKI. CVP should play a “limiting” rather than a target role within fluid resuscitation protocols [52]. Chen et al. found that early goal-directed diuretic therapy can improve the prognosis of sepsis [53]. In 105 patients with septic shock, Wang et al. showed that CVP was associated with kidney, liver, and lung function, sequential organ failure assessment scores, and lactate. Patients whose CVP remained below 8 mmHg during 7 days had a higher survival rate [54]. However, hypovolemia and renal hypoperfusion may occur in AKI patients if a too excessive fluid removal is pursued with diuretics or extracorporeal therapy [55].

Taken together, CVP plays an important role in the development of septic AKI by actively sustaining renal venous congestion and enhancing sepsis-related tissue edema.

### A high CVP should be avoided

**Fig. 1:**
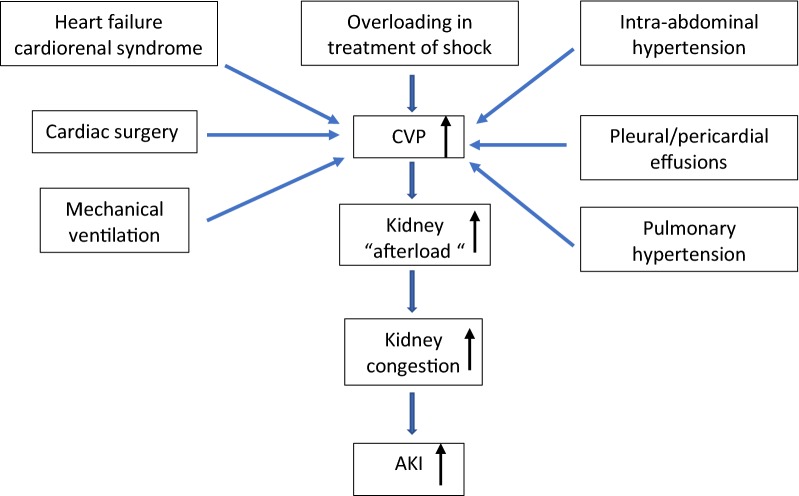
Relationship of all-caused high CVP and AKI. *CVP* central venous pressure, *AKI* acute kidney injury

Healthy persons have a low CVP [56]. A high CVP does not always signify fluid overload, yet may impede RBF return to the right atrium and increase the risk of AKI. Ventricular preload is determined by transmural pressure, which is the difference between intracardiac and extracardiac intrathoracic pressure. Changes in right or left ventricular compliance, pulmonary hypertension, pulmonary venous disease, chronic airway disease, positive pressure ventilation, cardiac tamponade, pleural effusion, and increased intra-abdominal pressure all can increase intrathoracic or pericardial pressure [57] and thus augment CVP, decrease venous return, and potentially injure the kidney. Any elevation or significant change in CVP may refer to either presence or severity of a particular disease process and its response to treatment (Fig. 1).

#### Intra-abdominal hypertension

Intra-abdominal hypertension is defined as an intra-abdominal pressure exceeding 1.6 kPa. Abdominal compartment syndrome is diagnosed when the intra-abdominal pressure persists above 2.7 kPa in association with new organ dysfunction or failure. Various diseases or conditions (e.g., pancreatitis, bile peritonitis, intra-abdominal hemorrhage, large abdominal masses, blunt abdominal trauma, recent abdominal surgery, …) but also ample fluid resuscitation may cause abdominal hypertension and abdominal compartment syndrome [58–61]. Sepsis is an important trigger of AKI in postoperative and trauma patients with intra-abdominal hypertension [62]. AKI related to high intra-abdominal pressure is mainly due to an increase in inferior vena cava and intrathoracic pressure resulting in CVP elevation. A close relationship exists between an increased intra-abdominal pressure and the presence of oliguria and a high serum creatinine. The impact of diuretics on CVP and recovery of renal function is limited. However, lowering intra-abdominal pressure decreased CVP, restored diuresis, and normalized serum creatinine levels [61]. In a swine model, elevated intra-abdominal pressure increased renal venous, pleural, wedge, and pulmonary artery pressures, whereas cardiac index and urine output decreased. Intravascular volume expansion significantly increased urine output [63]. Decreasing intra-abdominal pressure to offer more space for volume expansion may be the best option to lower CVP.

#### Cardiac surgery

The CVP recorded 6 h after elective or urgent coronary artery bypass grafting was a strong and independent predictor of mortality and AKI [64]. The risk-adjusted OR for AKI was 5.5 (95% CI 1.93, 15.5; *p* = 0.001) with every 5 mmHg rise in CVP for patients with a CVP < 9 mmHg. For patients with a CVP ≥ 9 mmHg at 6 h, risk-adjusted OR was 1.3 (95% CI 1.01, 1.65; *p* = 0.045) with every 5 mmHg rise in CVP [64]. Guinot et al. observed that renal dysfunction in cardiac surgery patients was associated with early postoperative vena cava dilatation and elevated CVP, secondary to an increase in right heart filling pressure due to impaired right ventricular diastolic function [65].

#### Mechanical ventilation

Mechanical ventilation, especially when combined with high positive end-expiratory pressure (PEEP), prone positioning, and lung recruitment maneuvers, induces a high CVP [57, 66, 67]. Lung recruitment decreased renal arterial blood flow and perfusion of renal cortex and medulla in both healthy pigs and in pigs with endotoxin-induced pulmonary arterial hypertension [66, 67]. A balance must be sought between adequate blood volume, lowest CVP, and lowest intrathoracic pressure by carefully titrating PEEP under hemodynamic monitoring [68].

#### Specific conditions

Pleural and pericardial effusions are often associated with an increase in CVP. Pleural or pericardial puncture and drainage will reduce CVP. An increased CVP is a hallmark of diseases accompanied by pulmonary hypertension. Specific treatments (e.g., inhaled nitric oxide) can decrease pulmonary pressure and CVP, yet may increase AKI risk [69]. The intrinsic response of renal vessels must thus always be weighed against the potential benefit of decreasing CVP when treating the primary disease.

### The “optimal” CVP should be personalized and kept as low as possible

Currently, no exact definition of “lowest possible CVP” can be given except that it should be a CVP that assures adequate cardiac output and preserves organ perfusion. It becomes evident that a personalized approach is needed to aim at the most optimal CVP. In different patient populations or cohorts of similar patients with different disease stages, this optimal CVP level also will be different. A retrospective analysis of more than 500,000 CVP recordings in more than 9000 patients showed that the highest quartile of mean CVP during the first 3 days [mean (SD); 17.4 (4.1) mmHg] was associated with a 33.6% higher adjusted risk of death as compared with the lowest quartile [7.4 (1.9) mmHg]. Poor secondary outcomes (i.e., prolonged mechanical ventilation or vasopressor use, longer ICU and hospital stay) were also associated with higher quartiles of elevated mean CVP. Prolonged duration of CVP > 10 mmHg was significantly higher in non-survivors [70]. Keeping CVP and fluid in balance is more challenging in patients exhibiting a high CVP but no volume overload. In addition, extracting volume is not always the best way to decrease CVP. Overzealous use of diuretics or excessive ultrafiltration may indeed cause unwarranted volume loss resulting in lower cardiac preload, CO, and RBF. Strict and continuous monitoring of cardiac output, CVP, and kidney perfusion is imperative to avoid under- or over-treatment [71, 72]. Patients with acute heart failure and a CVP < 10 cm H2O were more likely to develop worsening renal function within the first 24 h than those presenting with a CVP > 15 cm H2O [73]. This does not imply that a higher CVP must be targeted in this population but rather that a volume “deficit” due to excessive fluid restriction or elimination should absolutely be avoided. Any decision to lower CVP should be individualized. Improving lung–right heart interactions that sustain an elevated CVP in heart failure and cardiorenal syndrome appears to be more efficacious than reducing intravascular volume [26, 30].

## Conclusions

CVP is an innate pressure that is not only affected by manipulation of intravascular volume (fluid administration, restriction, or elimination) but also determined by various disease processes (intra-abdominal hypertension, pulmonary hypertension,…) or treatment (mechanical ventilation). Irrespective of volume status, an elevated CVP may harm the kidney by impeding renal venous return and causing renal interstitial edema. Individualizing CVP measurements and keeping CVP as low as possible should be encouraged to preserve kidney function or to avoid unnecessary renal damage.

## Abbreviations

AKI: acute kidney injury
CVP: central venous pressure
MAP: mean arterial pressure
RVP: renal venous pressure
RPP: renal perfusion pressure
CO: cardiac output
RBF: renal blood flow
MSFP: mean system filling pressure
EGDT: early goal-directed therapy
PEEP: positive end-expiratory pressure
